# Preconception mental health and developmental vulnerability at school entry: population-based cohort study

**DOI:** 10.1192/bjo.2026.11001

**Published:** 2026-03-23

**Authors:** Naomi N. Phagau, Paramdeep Kaur, Amanda S. Nitschke, Helena Abreu do Valle, Michael R. Law, Martin Guhn, Tim F. Oberlander, Gillian E. Hanley

**Affiliations:** Department of Gynaecology and Obstetrics, University of British Columbia, Vancouver, British Columbia, Canada; School of Population and Public Health, University of British Columbia, Vancouver, British Columbia, Canada; Centre for Health Services and Policy Research, University of British Columbia, Vancouver, British Columbia, Canada; Department of Pediatrics, University of British Columbia, Vancouver, British Columbia, Canada

**Keywords:** Depressive disorders, neurodevelopmental disorders, observational study, perinatal psychiatry, anxiety- or fear-related disorders

## Abstract

**Background:**

Perinatal depression and/or anxiety (depression–anxiety) have been associated with developmental disruptions. Less attention has been paid to preconception mental health, which could also contribute to adverse outcomes.

**Aims:**

To examine whether preconception mental health is associated with developmental vulnerability in children who were either exposed or unexposed to prenatal depression.

**Method:**

A population-based, retrospective cohort including 130 631 births to 108 340 pregnant people from British Columbia (Canada) between 1 January 2001 and 31 December 2012, with child development data in the form of the Early Development Instrument (EDI). Logistic regression using cluster-robust standard errors was used to compare the odds of vulnerability on EDI domains.

**Results:**

Children born to pregnant people in all groups with depression–anxiety preconception history were more likely to be considered vulnerable on all developmental domains, except for communication skills and general knowledge, than those without prenatal depression and no preconception depression–anxiety. After adjusting for confounders, effect size was largest for children born to a person with prenatal depression who had persistent depression–anxiety before they conceived on the domains of physical health and well-being (adjusted odds ratio 1.73 [95% CI: 1.56–1.92]). Children born to people with prenatal depression but no preconception depression–anxiety were probably more vulnerable on social competence and emotional maturity domains than those without prenatal depression and no preconception depression–anxiety.

**Conclusions:**

Preconception mental health is associated with child development, even after accounting for depression in pregnancy. We hypothesise that it is picking up on different experiences of mental illness through the life course and represents slightly different fetal exposures.

Pregnancy and the postpartum (perinatal) period are sensitive times in which detrimental exposures can have lasting effects on parents, infants and their families as a whole.^1–3^ Perinatal depression and anxiety have been associated with altered fetal programming and developmental disruptions.^4–6^ Whereas perinatal and postpartum mental health have been well studied, relatively less attention has been paid to parental mental health prior to conception, which could also contribute to adverse outcomes.^7^ There is considerable documental heterogeneity in the experience of depression,^8^ and it has been hypothesised that this is the end result of different pathophysiological pathways.^9^ The most important risk factor for perinatal depression is a history of depression, suggesting that the origins for prenatal depression (from conception to birth) often predate the pregnancy.^10^ Studies have also found that the person’s preconception mood remains relatively stable into the pregnancy, pointing to the importance of the preconception period in prenatal depression.^11^

Consideration of preconception mental health may allow us to account for some of the heterogeneity of depression. We hypothesise that different trajectories of prenatal depression may tap into slightly different exposures that could impact pregnancy, birth and infant outcomes differently.^11–13^ In a recent study, preconception mental health was found to alter the association between prenatal depression and/or anxiety (depression–anxiety) and gestational diabetes, such that only incident prenatal depression–anxiety was associated with increased risk for gestational diabetes.^14^ Poor preconception mental health has also been identified as an important risk factor for pregnancy complications, and as a potential risk factor for non-live births.^13^ An intergenerational cohort study conducted in Australia found that preconception mental health may be associated with the infant’s behavioural and emotional difficulties.^15^ We hypothesise that poor preconception metal health has lasting effects on the offspring, beyond infanthood, impacting on children’s neurobehavioural development.

The aim of this study was to examine whether preconception mental health is associated with developmental vulnerability by using the Early Development Instrument (EDI), a questionnaire that examines educational and behavioural development in the kindergarten. Importantly, vulnerability is associated with a higher risk for academic and behavioural challenges if subsequent support is not provided.^16^ Previous work has reported that fetal exposure to depression during pregnancy may predispose to childhood vulnerability on physical health and well-being and socio-emotional well-being, but that work did not examine whether preconception mental health interacts with prenatal depression to affect child development.^17^ Herein, we examine whether preconception depression–anxiety is associated with developmental vulnerability in children who were both exposed and unexposed to depression during fetal development.

## Method

### Study design

This population-based, retrospective cohort was created using birth data from British Columbia between 1 January 2001 and 31 December 2012, with EDI data up to 31 December 2018. The end of follow-up was defined to ensure inclusion of kindergarten data, because EDI is administered in the spring term. EDI data were linked to the British Columbia Perinatal Data Registry (BCPDR),^18^ the Medical Services Plan (MSP), the MSP Payment Information File, the Central Demographics File (previously known as the Consolidation File), the Discharge Abstract Database and British Columbia PharmaNet, which provided us with perinatal and out-patient physician visits, income, demographic, hospital admissions and medication data, respectively. Further information about these data-sets is available at the Population Data British Columbia’s project webpage: https://my.popdata.bc.ca/project_listings/20-167/collection_approval_dates. Access to data provided by data stewards is subject to approval, but can be requested for research projects through data stewards or their designated service providers. All inferences, opinions and conclusions drawn in this publication are those of the authors and do not reflect the opinions or policies of data stewards. The authors assert that all procedures contributing to this work comply with the ethical standards of the relevant national and institutional committees on human experimentation, and with the Helsinki Declaration of 1975 as revised in 2013. All procedures involving human subjects/patients were approved by the University of British Columbia Behavioural Research Ethics Board (no. H20-00345). Patient consent was not required for this study.

### Study cohort

Our cohort included children whose EDI was collected between 2006 and 2018. To ensure completeness of the data, we included children born in-province to a pregnant person registered for universal healthcare in British Columbia for at least 275 days during the year of, and prior to, their birth. We excluded pregnant people who had other mental health diagnoses 3 years preconception (Supplementary Table 1 available at https://doi.org/10.1192/bjo.2026.11001), and births with a final age of <23 weeks or >43 weeks, because these probably reflect data errors. Siblings and multiple pregnancies were not excluded.

### Exposure

**Fig. 1:**
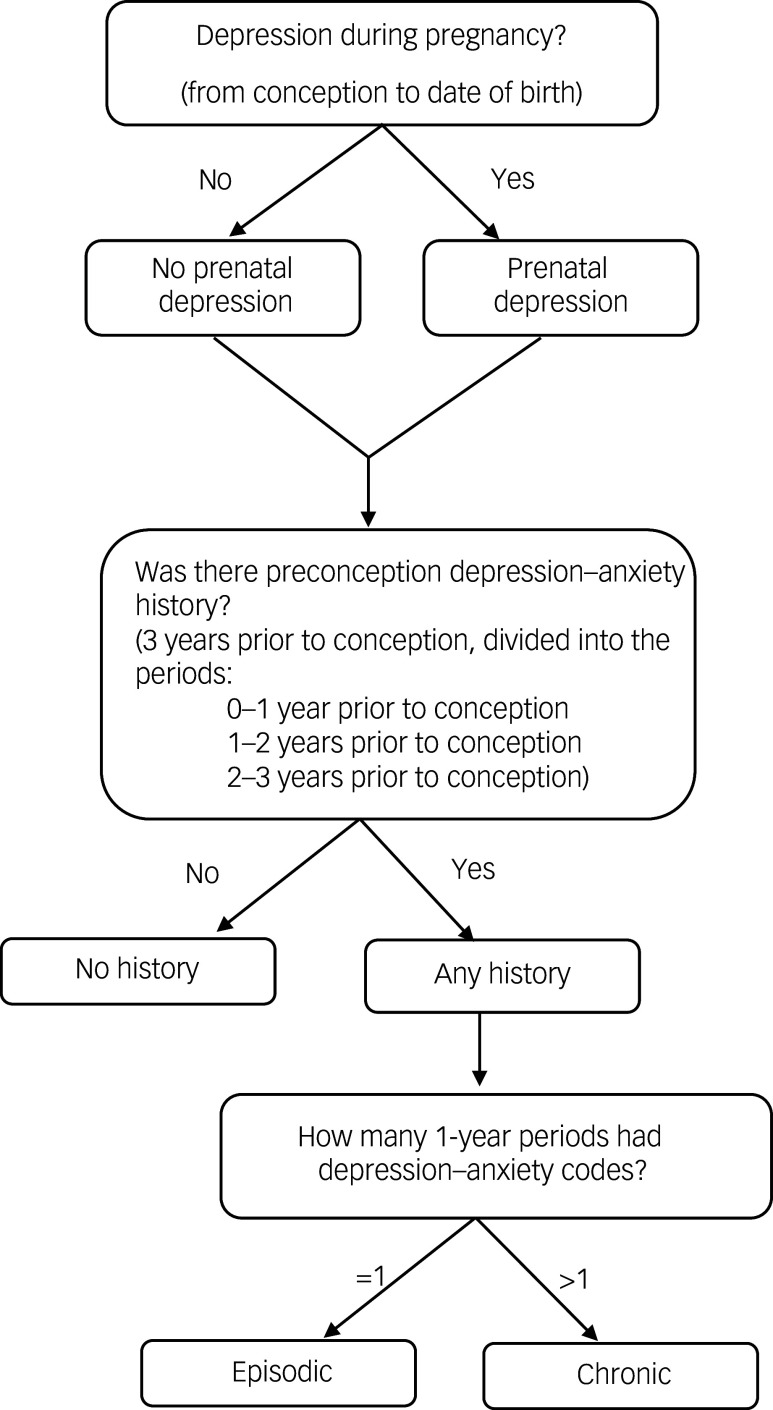
Decision tree for preconception depression and/or anxiety and prenatal depression duration classification. Depression–anxiety, depression and/or anxiety.

Mental health was identified using ICD-9 and ICD-10-CA diagnostic codes from physicians’ billing and hospital databases prior to conception and during pregnancy (Supplementary Table 2). We stratified pregnant people in our cohort into two groups based on their mental health status during pregnancy: (a) no prenatal depression and (b) prenatal depression diagnosis. We then examined their preconception mental health using previously created and validated variables that included anxiety, depression and an ICD-9 code used in British Columbia (50B) that healthcare providers can code when they see a patient with either anxiety or depression.^19^ We considered diagnostic codes present up to 3 years prior to their conception date, and we divided the periods into 0–1 year, 1–2 years and 2–3 years (Fig. 1). Both pregnancy mental health groups were further stratified into three groups according to their preconception mental health: (a) no history – the pregnant person did not have a single diagnostic code for depression–anxiety; (b) episodic – the pregnant person had one or more depression–anxiety diagnostic codes in only one preconception period; and (c) persistent – the pregnant person had one or more depression–anxiety diagnostic codes in more than one preconception period. These groups will be referred to as follows: no preconception history–no prenatal depression (reference group); episodic preconception–no prenatal depression; persistent preconception–no prenatal depression; no preconception history–prenatal depression; episodic preconception–prenatal depression; and persistent preconception–prenatal depression.

### Outcome

The EDI was developed to holistically measure children’s school readiness at a population level, and has been validated to assess child development in the early years.^20^ The EDI has been administered in waves throughout British Columbia every 3 years since 2001 (https://earlylearning.ubc.ca/). The waves capture subsets of the British Columbia population until all neighbourhoods are included. The EDI is a teacher-completed questionnaire that covers five developmental domains: physical health and well-being, social competence, emotional maturity, language and cognitive development, and communication skills and general knowledge. These domains have been identified as important areas of development and are indicative of subsequent success in school and beyond.^20–22^ Each domain has a total score ranging between 0 and 10, where 0 pertains to those children who are most vulnerable. Children deemed vulnerable on a domain are those in the bottom tenth percentile. School and teacher participation in the EDI is voluntary, and child participation occurs via passive consent. The EDI’s psychometric properties and validity have been well established, including examination of its associations with concurrent and later measures of developmental outcomes.^20,23,24^

### Covariates

We chose our covariates based on whether we thought they could be confounding the relationship between preconception depression–anxiety and child developmental vulnerability in the presence or absence of prenatal depression. We used sociodemographic data, including birth parent age at conception and neighbourhood income quintile (quintile 1 represents those with the lowest income, and quintile 5 includes those with the highest income); child biological gender, year of birth and data on pregnancy conditions such as parity (nulliparous or multiparous); pre-existing diabetes; and preconception body mass index (BMI). We also considered antidepressant, antipsychotic and anxiolytic use 1 year preconception. Date of conception was determined using the final gestational age from BCPDR, which is estimated using the last menstrual period, first ultrasound at <20 weeks gestation, clinical estimate from the newborn examination and documentation from the delivery chart. Parity is defined as the number of previous pregnancies delivered at ≥20 weeks of gestation and weighing at least 500 g. It is categorised as follows: nulliparous: mother has never delivered an infant weighing at least 500 g or with a gestational age of at least 20 weeks in a previous pregnancy; and multiparous: mother has previously carried a pregnancy resulting in a delivery of at least 500 g or 20 weeks’ gestation, regardless of the outcome.

### Statistical analysis

Demographic characteristics were compared between study groups using standardised mean differences (SMD), which are more appropriate for a cohort of this size because they quantify the magnitude of group differences independently of sample size and are consistently interpretable across continuous, binary and categorical variables.^25–28^ Developmental vulnerability was compared between our exposure groups using SMD. With respect to descriptive characteristics, we considered a difference between the groups to be meaningful when SMD exceeded 0.1, which is consistent with established methodological guidance.^25,29^

Logistic regression models using cluster-robust standard errors were run, and adjusted odds ratios (aOR) with confidence intervals were calculated to examine the relationship between pregnancy and preconception mental health and child vulnerability. We have missing data (0.5–2.0%) for some of our developmental domains, and these records were excluded when we ran the models. We also have missing data for covariate BMI (32.5%), income (1.7%) and parity (*n* = 6). We have used multiple imputation by chained equations, with 35 imputations from which estimates were pooled to impute the missing values of these variables. We also conducted the analysis following performing list-wise deletion of missing data. All statistical analysis was performed using RStudio statistical software (version 1.3.1093 for macOS; RStudio, PBC, Boston, Massachusetts, USA; https://posit.co/download/rstudio-desktop/) and Stata (version 16.1 for macOS; StataCorp LLC, College Station, Texas, USA; http://www.stata.com). All statistical tests were 2-sided, and statistical significance was defined as *P* < 0.05.

## Results

**Fig. 2:**
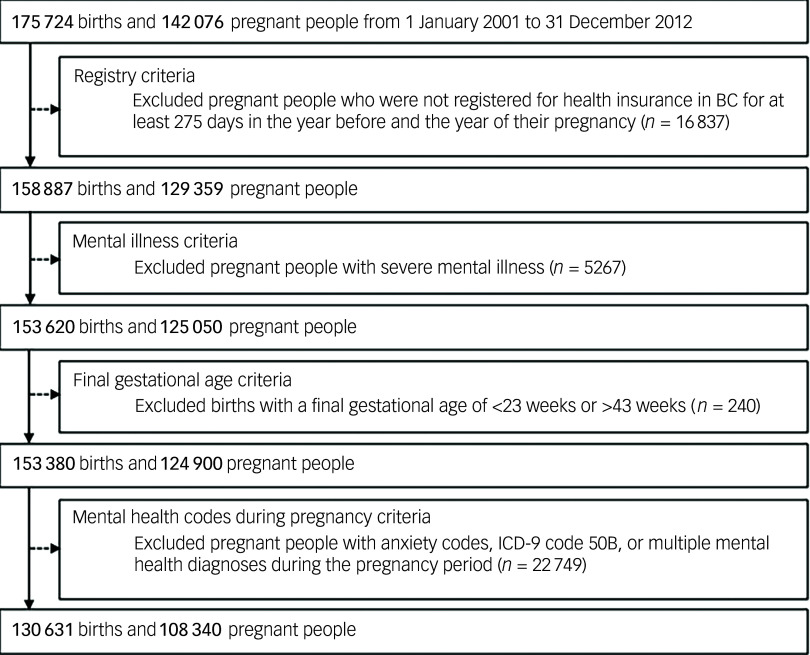
Participant flowchart for a population-based study examining preconception mental health and developmental vulnerability. BC, British Columbia.

Our original cohort included all children born in British Columbia but, because EDI data are administered in waves (and thus not population-based), we identified 175 724 births among 142 076 pregnant individuals (some individuals gave birth more than once in our study period), as shown in Fig. 2. Although 322 610 pregnancies were excluded due to lack of EDI data, both groups showed very similar values across many demographic and pregnancy characteristics (Supplementary Table 3). Following exclusion criteria, we included 130 631 births to 108 340 pregnant people. Our cohort included 66 652 children (51.0%) born to a pregnant person with no preconception history no prenatal depression; 40 369 children (30.9%) born to a pregnant person with episodic preconception–no prenatal depression; 17 521 children (13.4%) born to a pregnant person with persistent preconception–no prenatal depression; 1019 children (0.8%) born to a pregnant person with no preconception history–prenatal depression, and thus apparent incident depression in pregnancy; 2142 children (1.6%) born to a pregnant person with episodic preconception–prenatal depression; and 2928 children (2.2%) born to a pregnant person with persistent preconception–prenatal depression.

The mean age of the children in our cohort was 5.64 years. We did not observe large age differences between the no preconception history–no prenatal depression and episodic preconception–no prenatal depression groups (Table 1), but these were recorded for a few age categories when comparing the other groups with the no preconception history–no prenatal depression group. The no preconception history–no prenatal depression group was the least likely to use psychotropic drugs prior to conception, to smoke during pregnancy and to be multiparous. People in the persistent preconception–no prenatal depression, episodic preconception–prenatal depression and persistent preconception–prenatal depression groups were more likely to have had a hospital admission prior to birth than those in the no preconception history–no prenatal depression group. On average, children born to a pregnant person in the no preconception history–no prenatal depression group had the shortest stays in a level 3 neonatal intensive care unit.

**Table 1:** Characteristics by prenatal preconception mental health in full cohort (*N* = 130 631)

Variables	No preconception history–no prenatal depression (group A) *n* (%)	Episodic preconception history–no prenatal depression (group B) *n* (%)	SMD^a^ (A versus B)	Persistent preconception history–no prenatal depression (group C) *n* (%)	SMD^a^ (A versus C)	No preconception history–prenatal depression (group D) *n* (%)	SMD^a^ (A versus D)	Episodic preconception history–prenatal depression (group E) *n* (%)	SMD^a^ (A versus E)	Persistent preconception history–prenatal depression (group F) *n* (%)	SMD^a^ (A versus F)
Age at the time of conception, years											
<20	3415 (5.1)	1731 (4.3)	0.039	521 (2.9)	**0.109**	73 (7.1)	0.085	138 (6.4)	0.056	91 (3.1)	**0.101**
20–24	10 434 (15.6)	6493 (16.1)	0.012	2760 (15.7)	0.003	184 (18.0)	0.064	417 (19.5)	0.100	463 (15.8)	0.004
25–29	20 214 (30.3)	12 224 (30.3)	0.001	5269 (30.1)	0.005	285 (28.0)	0.052	577 (26.9)	0.075	844 (28.8)	0.033
30–34	21 538 (32.3)	12 940 (32.0)	0.005	5596 (32.0)	0.008	292 (28.7)	0.079	645 (30.1)	0.047	914 (31.2)	0.023
35–40	9458 (14.2)	5941 (14.7)	0.014	2804 (16.0)	0.051	167 (16.4)	0.061	313 (14.6)	0.012	529 (18.1)	**0.105**
≥40	1593 (2.4)	1040 (2.6)	0.012	571 (3.2)	0.052	18 (1.8)	0.044	52 (2.4)	0.002	87 (3.0)	0.036
Income quintile											
1 (lowest)	14 127 (21.6)	8492 (21.4)	0.004	3642 (21.1)	0.010	232 (23.3)	0.038	491 (23.3)	0.042	687 (23.8)	0.054
2	13 998 (21.4)	8306 (20.9)	0.011	3753 (21.7)	0.010	204 (20.5)	0.024	433 (20.6)	0.019	620 (21.5)	0.004
3	13 426 (20.5)	8290 (20.9)	0.010	3564 (20.6)	0.005	206 (20.7)	0.002	447 (21.2)	0.018	611 (21.2)	0.018
4	12 760 (19.5)	7909 (19.9)	0.011	3533 (20.5)	0.026	200 (20.1)	0.012	425 (20.2)	0.018	567 (19.6)	0.006
5 (highest)	11 074 (16.9)	6711 (16.9)	<0.001	2774 (16.1)	0.021	154 (15.5)	0.041	309 (14.7)	0.060	403 (14.0)	0.080
Unknown	1267 (1.9)	661 (1.6)	0.020	255 (1.5)	0.035	23 (2.3)	0.025	37 (1.7)	0.013	40 (1.4)	0.042
Preconception use of psychotropic drugs											
Antidepressant use	742 (1.11)	2840 (7.04)	**0.303**	3518 (20.1)	**0.648**	56 (5.50)	**0.247**	878 (41.0)	**1.121**	1912 (65.3)	**1.862**
Antipsychotic use	65 (0.10)	148 (0.37)	0.056	180 (1.03)	**0.125**	8 (0.79)	**0.104**	48 (2.24)	**0.200**	158 (5.40)	**0.329**
Anxiolytic use	1243 (1.86)	2263 (5.61)	**0.198**	2101 (12.0)	**0.407**	48 (4.71)	**0.160**	327 (15.27)	**0.493**	738 (25.2)	**0.726**
Pregnancy conditions and risk factors											
Current smoker	5276 (7.9)	4678 (11.6)	**0.124**	2585 (14.8)	**0.217**	141 (13.8)	**0.191**	312 (14.6)	**0.212**	577 (19.7)	**0.347**
Parity											
Multiparous	34 517 (51.8)	24 162 (59.9)	**0.163**	11577 (66.1)	**0.294**	535 (52.5)	0.014	1298 (60.6)	**0.178**	1902 (65.0)	**0.270**
Nulliparous	32 132 (48.2)	16 205 (40.1)	**0.163**	5943 (33.9)	**0.294**	484 (47.5)	0.014	844 (39.4)	**0.178**	1026 (35.0)	**0.270**
Unknown	≤5	≤5	0.001	≤5	0.002	0 (0)	0.009	0 (0.0)	0.009	0 (0)	0.009
Previous preterm deliveries	2095 (3.1)	1848 (4.6)	0.075	1050 (6.0)	**0.137**	36 (3.5)	0.022	104 (4.9)	0.088	192 (6.6)	**0.159**
Preconception BMI^b^											
<18.5 (underweight)	2528 (3.8)	1291 (3.2)	0.032	506 (2.9)	0.050	39 (3.8)	0.002	74 (3.5)	0.018	91 (3.1)	0.038
18.5–24.99 (normal)	28 226 (42.3)	16 059 (39.8)	0.052	6447 (36.8)	**0.114**	406 (39.8)	0.051	787 (36.7)	**0.115**	1002 (34.2)	**0.168**
25.0–29.99 (overweight)	9170 (13.8)	6118 (15.2)	0.040	2725 (15.6)	0.051	162 (15.9)	0.060	352 (16.4)	0.075	497 (17.0)	0.089
≥30 (obese)	5203 (7.8)	3819 (9.5)	0.059	1987 (11.3)	**0.120**	91 (8.9)	0.041	226 (10.6)	0.095	407 (13.9)	**0.197**
Missing	21 525 (32.3)	3082 (32.4)	0.002	5856 (33.4)	0.024	321 (31.5)	0.017	703 (32.8)	0.011	931 (31.8)	0.011
Diabetes											
Any type of diabetes prior to delivery	4772 (7.2)	2964 (7.3)	0.007	1427 (8.1)	0.037	81 (7.9)	0.030	150 (7.0)	0.006	243 (8.3)	0.043
Gestational diabetes	4490 (6.7)	2768 (6.9)	0.005	1325 (7.6)	0.032	74 (7.3)	0.021	133 (6.2)	0.021	224 (7.7)	0.035
Diabetes pre-pregnancy	282 (0.4)	196 (0.5)	0.009	102 (0.6)	0.022	7 (0.7)	0.036	17 (0.8)	0.048	19 (0.6)	0.031
Hypertension											
Pregnancy-induced hypertension	3521 (5.3)	2156 (5.3)	0.003	1011 (5.8)	0.021	52 (5.1)	0.008	127 (5.9)	0.028	219 (7.5)	0.090
Hypertension due to other causes	1999 (3.0)	1226 (3.0)	0.002	572 (3.3)	0.015	32 (3.1)	0.008	71 (3.3)	0.018	121 (4.1)	0.061
Labour, delivery and antenatal care characteristics											
Antenatal care, mean (s.d.)											
Number of antenatal visits	9.31 (2.91)	9.40 (3.01)	0.032	9.53 (3.18)	0.073	9.53 (3.14)	0.076	9.55 (3.04)	0.081	9.68 (3.46)	**0.118**
Prior hospital admission	0.11 (0.47)	0.14 (0.54)	0.054	0.18 (0.65)	**0.119**	0.14 (0.49)	0.065	0.21 (0.63)	**0.165**	0.24 (0.63)	**0.228**
Mode of delivery											
Emergency CS	12291 (18.4)	7576 (18.8)	0.008	3319 (18.9)	0.013	195 (19.1)	0.018	390 (18.2)	0.006	610 (20.8)	0.060
Elective CS	7249 (10.9)	4965 (12.3)	0.044	2428 (13.8)	0.091	113 (11.1)	0.007	304 (14.2)	0.100	392 (13.4)	0.077
Spontaneous vaginal birth	39 796 (59.7)	24 070 (59.6)	0.002	10 277 (58.6)	0.021	599 (58.8)	0.019	1276 (59.6)	0.003	1670 (57.0)	0.054
Instrumental vaginal birth	7316 (11.0)	3758 (9.3)	0.055	1497 (8.5)	0.082	112 (11.0)	<0.001	172 (8.0)	**0.101**	256 (8.7)	0.075
Induced labour	13 969 (21.0)	8548 (21.2)	0.005	3814 (21.8)	0.020	222 (21.8)	0.020	444 (20.7)	0.006	637 (21.8)	0.020
Antibiotics used during labour and delivery	25 440 (38.2)	16 240 (40.2)	0.042	7321 (41.8)	0.074	401 (39.4)	0.024	859 (40.1)	0.040	1247 (42.6)	0.090
Infant and child characteristics											
Biological gender											
Female	32 519 (48.8)	19 594 (48.5)	0.005	8557 (48.8)	0.001	484 (47.5)	0.026	1047 (48.9)	0.002	1425 (48.7)	0.002
Male	34 132 (51.2)	20 775 (51.5)	0.005	8964 (51.2)	0.001	535 (52.5)	0.026	1095 (51.1)	0.002	1503 (51.3)	0.002
Other	≤5	0	0.005	0	0.005	0	0.005	0	0.005	0	0.005
Year of birth											
2001–2003	17 511 (26.3)	8239 (20.4)	**0.139**	2159 (12.3)	**0.359**	274 (26.9)	0.014	595 (27.8)	0.034	396 (13.5)	**0.323**
2004–2006	19 105 (28.6)	11 130 (27.6)	0.024	4819 (27.5)	0.026	291 (28.5)	0.002	614 (28.7)	<0.0001	794 (27.1)	0.034
2007–2009	16 124 (24.2)	10 835 (26.8)	0.061	5333 (30.4)	**0.140**	236 (23.2)	0.024	513 (23.9)	0.006	864 (29.5)	**0.120**
2010–2012	13 912 (20.9)	10 165 (25.2)	**0.102**	5210 (29.7)	**0.205**	218 (21.4)	0.013	420 (19.6)	0.031	874 (29.8)	**0.207**
Gestational age at birth, weeks, mean (s.d.)	38.79 (1.82)	38.64 (1.93)	0.079	38.51 (1.95)	**0.145**	38.66 (1.92)	0.068	38.55 (1.9)	**0.126**	38.33 (2.11)	**0.230**
Admission to NICU	2824 (4.2)	2026 (5.0)	0.037	1049 (6.0)	0.080	54 (5.3)	0.050	97 (4.5)	0.014	226 (7.7)	**0.147**
Number of days in NICU, mean (s.d.)											
Level II	5.65 (9.60)	6.79 (10.95)	**0.110**	7.23 (11.55)	**0.154**	6.44 (11.28)	0.084	7.29 (9.72)	**0.182**	8.05 (11.87)	**0.233**
Level III	3.66 (14.22)	5.16 (13.61)	**0.154**	4.57 (11.71)	**0.117**	7.33 (19.93)	**0.265**	3.51 (8.74)	0.033	6.54 (14.88)	**0.268**
Baby birth weight, mean (s.d.)	3424.69 (546.80)	3427.89 (569.16)	0.006	3417.50 (577.7)	0.013	3414.30 (551.8)	0.019	3424.55 (555.8)	<0.001	3382.06 (596.2)	0.075
Small for gestational age^c^	4411 (6.6)	2346 (5.8)	0.033	985 (5.6)	0.42	60 (5.9)	0.030	116 (5.4)	0.051	180 (6.1)	0.019
Large for gestational age^d^	8446 (12.7)	5733 (14.2)	0.045	2652 (15.1)	0.071	125 (12.3)	0.012	314 (14.7)	0.058	442 (15.1)	0.070
Apgar score at 5 min <7	804 (1.2)	556 (1.4)	0.015	285 (1.6)	0.036	17 (1.7)	0.039	40 (1.9)	0.017	93 (3.2)	**0.135**
Baby congenital malformation	3090 (4.6)	1842 (4.6)	0.003	804 (4.6)	0.002	49 (4.8)	0.008	92 (4.3)	0.017	145 (5.0)	0.015

SMD, standardised mean difference; BMI, body mass index; CS, Caesarean section; NICU, neonatal intensive care unit.

a. SMDs shown in bold represent meaningful differences between groups (SMD > 0.10).

b. Weight (kg) divided by height (m) squared.

c. Below the tenth percentile of weight for final gestational age and biological gender, according to charts used in British Columbia, Canada.

d. Above the 90th percentile of weight for final gestational age and biological gender, according to charts used in British Columbia, Canada.

The proportions of developmental vulnerability among our exposure groups are presented in Table 2. Compared with children born to a pregnant person in the group with no preconception history–no prenatal depression, those born to someone in the persistent preconception–no prenatal depression, episodic preconception–prenatal depression and persistent preconception–prenatal depression groups had greater vulnerabilities on all domains, except for communication skills and general knowledge. Children born to a person in the no preconception history–prenatal depression group had greater vulnerability on the social competence domain than children born to a pregnant person in the group with no preconception history–no prenatal depression.

**Table 2:** Frequency of developmental vulnerability by prenatal and preconception mental health for full cohort (*N* = 130 631)

Developmental domain	*n* (%)	No preconception history–no prenatal depression (group A) *n* (%)	Episodic preconception history–no prenatal depression (group B) *n* (%)	SMD^a^ (A versus B)	Persistent preconception history–no prenatal depression (group C) *n* (%)	SMD^a^ (A versus C)	No preconception history–prenatal depression (group D) *n* (%)	SMD^a^ (A versus D)	Episodic preconception history–prenatal depression(group E) *n* (%)	SMD^a^ (A versus E)	Persistent preconception history–prenatal depression (group F) *n* (%)	SMD^a^ (A versus F)
Physical health and well-being	129 798 (99.4)	7575 (11.4)	5583 (13.9)	0.075	2912 (16.7)	**0.152**	130 (12.8)	0.043	378 (17.8)	**0.180**	634 (21.9)	**0.282**
Social competence	129 763 (99.3)	7922 (12.0)	5631 (14.0)	0.062	2854 (16.4)	**0.127**	157 (15.5)	**0.104**	366 (17.2)	**0.149**	610 (21.0)	**0.245**
Emotional maturity	129 227 (98.9)	7926 (12.0)	5662 (14.2)	0.064	2811 (16.2)	**0.120**	151 (15.0)	0.086	330 (15.6)	**0.103**	609 (21.1)	**0.245**
Language and cognitive development	129 246 (98.9)	5219 (7.9)	3829 (9.6)	0.059	1888 (10.9)	**0.101**	100 (10.0)	0.072	238 (11.2)	**0.113**	373 (12.9)	**0.164**
Communication skills and general knowledge	129 877 (99.4)	7871 (11.9)	4671 (11.6)	0.007	2109 (12.1)	0.007	136 (13.4)	0.046	272 (12.8)	0.027	404 (13.9)	0.060
≥1 domain	128 351 (98.2)	17 490 (26.7)	11 674 (29.4)	0.060	5651 (32.7)	**0.132**	305 (30.5)	0.084	692 (32.9)	**0.136**	1125 (39.3)	**0.269**
≥2 domains	128 351 (98.2)	9327 (14.2)	6590 (16.6)	0.066	3331 (19.3)	**0.135**	176 (17.6)	0.092	417 (19.8)	**0.149**	681 (23.7)	**0.244**

a. Standardised mean differences (SMDs) shown in bold represent meaningful differences between groups (SMD > 0.10).

A child is deemed to be vulnerable on a particular domain if their scores are in the bottom 10% using British Columbia-Provincial cut-offs.

Mean scores on each developmental domain are presented in Table 3, and they show a pattern similar to the vulnerability proportions. Children born to a person in the persistent preconception–prenatal depression group had the lowest mean domain scores, with SMD > 0.1 in all domains compared with the group with no preconception history–no prenatal depression.

**Table 3:** Mean scores for each Early Developmental Instrument domain by prenatal and preconception mental health for full cohort (*N* = 130 631)

Developmental domain	Children included, *n* (%)	No preconception history–no prenatal depression (group A), mean (s.d.)	Episodic preconception history–no prenatal depression (group B), mean (s.d.)	SMD^a^ (A versus B)	Persistent preconception history–no prenatal depression (group C), mean (s.d.)	SMD^a^ (A versus C)	No preconception history–prenatal depression (group D), mean (s.d.)	SMD^a^ (A versus D)	Episodic preconception history–prenatal depression (group E), mean (s.d.)	SMD^a^ (A versus E)	Persistent preconception history–prenatal depression (group F), mean (s.d.)	SMD^a^ (A versus F)
Physical health and well-being	129 798 (99.4)	8.51 (1.46)	8.36 (1.54)	**0.103**	8.20 (1.62)	**0.202**	8.36 (1.55)	0.099	8.16 (1.68)	**0.222**	7.96 (1.71)	**0.350**
Social competence	129 763 (99.3)	8.22 (1.89)	8.06 (1.97)	0.083	7.90 (2.04)	**0.163**	7.97 (2.03)	**0.126**	7.87 (2.07)	**0.178**	7.60 (2.17)	**0.304**
Emotional maturity	129 227 (98.9)	7.87 (1.59)	7.75 (1.67)	0.074	7.62 (1.73)	**0.147**	7.71 (1.67)	0.098	7.64 (1.73)	**0.139**	7.34 (1.85)	**0.304**
Language and cognitive development	129 246 (98.9)	8.42 (1.76)	8.28 (1.87)	0.077	8.16 (1.94)	**0.141**	8.26 (1.94)	0.089	8.13 (1.97)	**0.157**	7.97 (2.04)	**0.239**
Communication skills and general knowledge	129 877 (99.4)	7.40 (2.68)	7.36 (2.66)	0.016	7.29 (2.66)	0.044	7.24 (2.74)	0.062	7.24 (2.72)	0.059	7.08 (2.74)	**0.118**

a. Standardised mean differences (SMDs) shown in bold represent meaningful differences between groups (SMD > 0.10).

Mean domain scores range from 0 to 10, with 10 being the highest score indicating the most positive, desirable development.

### Unadjusted models

Compared with the no preconception history–no prenatal depression group, children born to pregnant people in all groups with depression–anxiety preconception history (episodic preconception–no prenatal depression, persistent preconception–no prenatal depression, episodic preconception–prenatal depression and persistent preconception–prenatal depression) were more likely to be considered vulnerable on all developmental domains, except for communication skills and general knowledge (Table 4). They were also more likely to be vulnerable in one or more domains and two or more domains. Greater odds of vulnerability were observed in the persistent preconception–prenatal depression group on the physical health and well-being domain (odds ratio 2.16 [95% CI: 1.98–2.37]). Children born to pregnant people in the no preconception history–prenatal depression group were more likely to be vulnerable on all domains, except for physical health and well-being and communication skills and general knowledge.

**Table 4:** Multivariable logistic regression models: prenatal and preconception mental health and sub-domain vulnerability based on imputed data-sets

Domains/sub-domains	Episodic preconception history–no prenatal depression		Persistent preconception history–no prenatal depression		No preconception history–prenatal depression		Episodic preconception history–prenatal depression		Persistent preconception history–prenatal depression	
Developmental domain	Crude OR (95% CI)^a^	Adjusted OR (95% CI)^a^^,^^b^	Crude OR (95% CI)^a^	Adjusted OR (95% CI)^a^^,^^b^	Crude OR (95% CI)^a^	Adjusted OR (95% CI)^a^^,^^b^	Crude OR (95% CI)^a^	Adjusted OR (95% CI)^a^^,^^b^	Crude OR (95% CI)^a^	Adjusted OR (95% CI)^a^^,^^b^
Physical health and well-being	**1.25** **(1.20–1.30)**	**1.19** **(1.14–1.23)**	**1.55** **(1.48–1.63)**	**1.40** **(1.33–1.47)**	1.14 (0.94–1.37)	1.04 (0.86–1.26)	**1.67** **(1.49– 1.88)**	**1.40** **(1.24–1.59)**	**2.16** **(1.98– 2.37)**	**1.73** **(1.56–1.92)**
Social competence	**1.20** **(1.16–1.25)**	**1.18** **(1.13–1.22)**	**1.44** **(1.37–1.51)**	**1.38** **(1.31–1.45)**	**1.35** **(1.14–1.61)**	**1.27** **(1.07–1.52)**	**1.53** **(1.36–1.72)**	**1.36** **(1.20–1.54)**	**1.95** **(1.78–2.14)**	**1.70** **(1.53–1.89)**
Emotional maturity	**1.21** **(1.16–1.25)**	**1.18** **(1.13–1.22)**	**1.41** **(1.35–1.48)**	**1.33** **(1.27–1.40)**	**1.29** **(1.08–1.53)**	**1.22** **(1.02– 1.45)**	**1.35** **(1.20– 1.52)**	**1.22** **(1.07–1.38)**	**1.95** **(1.78–2.14)**	**1.67** **(1.50–1.86)**
Language and cognitive development	**1.23** **(1.18–1.29)**	**1.18** **(1.12–1.23)**	**1.42** **(1.34–1.50)**	**1.31** **(1.23–1.40)**	**1.29** **(1.04–1.58)**	1.19 (0.96– 1.47)	**1.47** **(1.28– 1.69)**	**1.22** **(1.05– 1.42)**	**1.72** **(1.54– 1.93)**	**1.45** **(1.27–1.64)**
Communication skills and general knowledge	0.98 (0.94–1.01)	**0.93** **(0.90–0.97)**	1.02 (0.97–1.08)	0.95 (0.90–1.00)	1.15 (0.96–1.38)	1.08 (0.89–1.30)	1.09 (0.95– 1.24)	0.95 (0.83–1.09)	**1.19** **(1.07–1.33)**	1.03 (0.92–1.16)
≥1 domain	**1.14** **(1.11– 1.18)**	**1.11** **(1.07–1.14)**	**1.33** **(1.28 –1.38)**	**1.25** **(1.20–1.30)**	**1.20** **(1.05–1.38)**	1.13 (0.98–1.30)	**1.34** **(1.22–1.47)**	**1.17** **(1.06–1.30)**	**1.77** **(1.64–1.91)**	**1.50** **(1.38–1.64)**
≥2 domains	**1.20** **(1.16–1.24)**	**1.16** **(1.11–1.20)**	**1.44** **(1.37–1.50)**	**1.34** **(1.28–1.41)**	**1.29** **(1.09–1.52)**	**1.20** **(1.01–1.42)**	**1.49** **(1.33–1.66)**	**1.29** **(1.15–1.46)**	**1.87** **(1.71–2.05)**	**1.59** **(1.43–1.76)**

OR, odds ratio.

a. Values in bold indicate significance at *P* < 0.05.

b. Adjusted for the pregnant person’s characteristics at conception (age at the time of conception and income quintile), child biological gender, year of birth, nulliparity, pre-existing diabetes, preconception body mass index and medication use (1-year preconception use of antidepressants, antipsychotics or anxiolytics).

A child is deemed to be vulnerable on a particular domain if their scores are in the bottom 10% using BC-Provincial cut-offs.

Reference group refers to those without prenatal depression and without diagnosed depression and/or anxiety 0–3 years preconception.

### Adjusted models

The offspring of all groups, apart from the no preconception history–prenatal depression group, were more likely to be vulnerable on all domains, except for language and cognitive development (aOR 1.19 [95% CI: 0.96–1.47]) and communication skills and general knowledge (aOR 1.08 [95% CI: 0.89–1.30]) compared with the no preconception history–no prenatal depression group (Table 4). Children born to people in the no preconception–prenatal depression group were likely to be more vulnerable than the reference group on the social competence (aOR 1.27 [95% CI: 1.07–1.52]) and emotional maturity domains (aOR 1.22 [95% CI: 1.02–1.45]), and on two or more domains (aOR 1.20 [95% CI: 1.01–1.42]). A negative association was found for children born to people with episodic preconception–no prenatal depression, who were less likely to be vulnerable on the communication and general knowledge domain than the no preconception history–no prenatal depression group (aOR 0.93 [95% CI: 0.90–0.97]). The results of the analysis conducted following list-wise deletion of missing data are presented in Supplementary Table 4.

## Discussion

In this retrospective, population-based study in British Columbia, Canada, we found associations between different types of preconception depression–anxiety histories and developmental vulnerability within a cohort of pregnant people with and without a gestational diagnosis of depression. Children born to a pregnant person with prenatal depression and who had episodic or persistent preconception depression–anxiety appeared to be at higher risk for developmental vulnerability compared with those born to a pregnant person with no prenatal depression and no preconception depression–anxiety history. The associations differed in effect size based on duration of preconception depression and anxiety, with higher likelihoods of being considered developmentally vulnerable on physical health and well-being, social competence, emotional maturity, language and cognitive development being associated with a persistent history of depression–anxiety. Importantly, those born to a pregnant person without prenatal depression diagnoses, but who had episodic or persistent preconception depression–anxiety, were also more likely to be vulnerable than children born to a pregnant person with no prenatal depression and no preconception depression–anxiety history. Moreover, incident depression during pregnancy (prenatal depression but no preconception depression–anxiety history) was associated with vulnerability on fewer domains than any history of preconception depression–anxiety.

Our findings from this analysis align with previous literature suggesting that preconception mental health conditions may influence infant and neonatal outcomes,^8,13,30,31^ and suggest that there are lasting effects into early childhood. These findings may be reflective of the fact that persistent exposure to poor preconception mental health could alter a pregnant person’s ability to respond to stress during the pregnancy period, which we know also independently leads to disruption of neurobehavioural development.^13^ Additionally, poor preconception mental health is associated with prenatal and postpartum mental health.^32^ The cumulative effects of preconception, prenatal and postpartum stress may all contribute to developmental vulnerability, pointing to a need for intervention and prevention as early as possible, and even prior to conception. Whereas there has been increased awareness of the need for preconception mental health screening and intervention, it is still often not accounted for in preconception healthcare and the best approach to deliver preconception mental healthcare has not been determined.^33,34^ Our findings suggest that addressing mental health concerns in the preconception period may be a more appropriate time, especially when considering the more pronounced effects of pre-existing depression, particularly persistent depression, on child development.

It should be noted that, whereas we designed our preconception depression variable as an attempt to differentiate between alternative phenotypes of depression, it may be inadvertently picking up the severity of depression. For example, some of the pregnant people who had their first diagnostic code for depression during pregnancy may have been suffering from mild depression for many years and, through routine screening in pregnancy, it was first noticed during pregnancy whereas those with more severe depression may have been included in the persistent group. This hypothesis might also be supported by our finding of a protective effect for communication skills and general knowledge in those with preconception depression–anxiety but without prenatal depression. It is possible that this group represents individuals who are euthymic during their pregnancy because of their prior successful treatment of depression–anxiety. It is possible that communication skills and general knowledge are developmental domains that are less vulnerable to prenatal depression. Despite this limitation, our findings suggest that preconception mental health is valuable in better delineation of fetal exposure to prenatal depression. There were important differences found in child development based on preconception mental health, suggesting that preconception mental health matters.

Our study has many strengths, including the use of population-based data that included all births in British Columbia and having EDI records, which limited the likelihood of ascertainment bias. The use of EDI allowed us access to high-quality data covering multiple areas of development. Kindergarten teachers are believed to provide meaningful and reliable information through EDI, due to their training in child development and behaviour in early learning settings and awareness of children’s ranging academic skills.^35^ There is also very little likelihood that a kindergarten teacher would be aware of a child’s exposure to depression *in utero*, and thus there is little to no possibility of bias. Moreover, we were able to collect outcome information regardless of children’s contact with the healthcare system, which is potentially influenced by parents’ mental health and healthcare-seeking behaviour, and by socioeconomic vulnerability. Our access to longitudinal data is also a strength, because there are limited existing studies with the data necessary to incorporate preconception mental health into studies of child educational and behavioural outcomes.

However, our study has important limitations, including those introduced through the use of population-based administrative data-sets. For example, while we included income quintile in our models, we were missing important information on ethnicity, race, education level and family environment. Studies have reported that familial context and parental hostile behaviour are important factors in the relationship between prenatal depression and child development.^36,37^ It is likely that socioeconomic vulnerability and family functioning are also relevant for the association between preconception mental health and child development, and our study may suffer from residual confounding. We were also unable to account for genetic predisposition of mental health conditions in our analysis. Our decision to focus on those without severe illness and to exclude those with other forms of mental illness was made to provide a more precise estimate of the association between preconception depression–anxiety in those with and without perinatal depression. However, this does mean that our findings are not generalisable to those populations. Lastly, we were unable to factor in additional psychosocial services that a pregnant person may use to treat and address their mental health concerns, such as cognitive–behavioural therapy provided by a psychologist, because these services are not included in British Columbia’s medical service plan.

Overall, our study shows that poor preconception mental health may influence physical health and well-being and socio-emotional development in early childhood. These findings align with previous research associating preconception mental health history with numerous pregnancy and infant outcomes, including increased risk for gestational diabetes mellitus;^14^ increased risk for low birth weight and pregnancy complications, including premature labour, low-lying placenta and high blood pressure;^13^ and higher levels of behavioural problems and emotional reactivity in infanthood.^38,39^ These findings may be used to identify potentially vulnerable children before they develop a psychiatric disorder, and to allow for earlier interventions to meet their unmet needs. Clinically, these results suggest the need for greater support and treatment for mental illness prior to pregnancy, as well as the importance of considering mental health history during pregnancy and postpartum.

## Supporting information

Phagau et al. supplementary material Phagau et al. supplementary material

## Data Availability

Access to data provided by the data stewards is subject to approval, but can be requested for research projects through the data stewards or their designated service providers.

## Supplementary material

The supplementary material is available online at https://doi.org/10.1192/bjo.2026.11001
