# Supplementary material for: Preconception mental health and developmental vulnerability at school entry: population-based cohort study
Source: BJPsych Open. 2026 Mar 23;12(2):e90. doi: 10.1192/bjo.2026.11001 (PMC13107315; doi:10.1192/bjo.2026.11001)
Supplement: Phagau et al. supplementary material [file S2056472426110011sup001.docx]

**Supplemental Table 1.** Excluded mental health conditions and corresponding diagnostic codes. MSP primarily uses the ICD-9-CM system of diagnostic codes, supplemented by a set of BC specific codes (*). DAD uses primarily ICD-10 diagnostic codes.

| **Diagnostic exclusion** | **Descriptor or subtype** | **MSP diagnostic code** | **DAD diagnostic code** |
| --- | --- | --- | --- |
| Schizophrenia | -- | 295.xx | F20.xx |
|  | Schizotypal disorder | 295.6 | F21.xx |
|  | Schizoaffective disorder | 295.7 | F25.xx |
| Delusional and/or psychotic disorders | -- | 297.xx | F22.xx |
|  | Brief | 293.81, 293.82, 298.3, 298.4, 298.8 | F23.xx |
|  | Shared | 297.3 | F24.xx |
|  | Other | 298.1 | F28.xx |
|  | Unspecified | 298.9 | F29.xx |
| Manic affective disorder | Recurrent episode | 296.1 | F30.xx |
| Bipolar disorders | -- | 296.xx (excluding 296.2, 296.3, and 296.9) | F31.xx |

Abbreviations: MSP, Medical Services Plan; ICD, International Classification of Diseases; BC, British Columbia; DAD, Discharge Abstract Database.

**Supplemental Table 2.** List of relevant health conditions and corresponding diagnostic codes. MSP primarily uses the ICD-9-CM system of diagnostic codes, supplemented by a set of BC specific codes (*). DAD uses primarily ICD-10 diagnostic codes.

| **Diagnostic inclusion** | **Descriptor or subtype** | **MSP diagnostic code** | **DAD diagnostic code** |
| --- | --- | --- | --- |
| **Depressive diagnoses^a^** | | | |
| Depressive disorder |  | 311.x | -- |
| Major depressive disorder | Single episode | 296.2 | F32.xx |
|  | Recurrent | 296.3 | F33.xx |
| Persistent depressive disorder | Dysthymic disorders | 300.4x | F34.1, F34.8, F34.9 |
| Psychosis | Depressive type | 298.0x | -- |
| Adjustment disorder or severe stress reaction | Adjustment disorder with depression | 309.0, 309.1 | F43.21 |
|  | Adjustment disorder with depression and anxiety | -- | F43.23 |
| **Anxiety diagnoses^b^** | | | |
| Anxiety disorders |  | 300.0x | F41.xx |
| Adjustment disorder or severe stress reaction | Posttraumatic stress disorder | 309.81 | F43.1x |
|  | Adjustment disorder with anxiety | 309.2x | F43.22 |
| Phobic disorders | None | 300.2x | F40.xx |
| Obsessive compulsive disorders |  | 300.3x | F42.xx |
| **Other diagnoses^b^** | | | |
| Depression or anxiety |  | 50B* | -- |

Abbreviations: MSP, Medical Services Plan; ICD, International Classification of Diseases; BC, British Columbia; DAD, Discharge Abstract Database. ^a^ prenatal and preconception period; ^b^ preconception period

**Supplementary Table 3**. Characteristics by **Early Development Instrument** within a cohort of births between January 01, 2001 and January 31, 2012; excluding pregnant people with a severe mental illness diagnosis (schizophrenia, delusional and/or psychotic disorders, manic affective disorder, and/or bipolar disorder) and/or a final gestational age of less than 23 weeks or more than 43 weeks (n=492,409)

| **Early Developmental Instrument** | | |
| --- | --- | --- |
|  | No N = 322,610 | Yes N = 169,799 |
| **Pregnant Parent Sociodemographic Characteristics** | | |
| **Age Categories, n (%)** |  |  |
| < 20 years | 9936 (3.1) | 6000 (3.5) |
| 20 – 24 years | 42,943 (13.3) | 25,584 (15.1) |
| 25-29 years | 89,194 (27.6) | 48,428 (28.5) |
| 30-34 years | 107,029 (33.2) | 54,740 (32.2) |
| 35-39 years | 60,103 (18.6) | 28,916 (17.0) |
| ≥40 years | 13,405 (4.2) | 6131 (3.6) |
| **Income quintile, n (%)** |  |  |
| 1 | 70,398 (21.8) | 36,885 (21.7) |
| 2 | 66,975 (20.8) | 35,658 (21.0) |
| 3 | 62,530 (19.4) | 33,913 (20.0) |
| 4 | 60,442 (18.7) | 32,316 (19.0) |
| 5 | 50,243 (15.6) | 27,178 (16.0) |
| Unknown/Missing | 12,022 (3.7) | 3849 (2.3) |
| **Current smoker, n (%)** | 27,738 (8.6) | 18,649 (11.0) |
| **Preconception BMI, n (%) ^a^** |  |  |
| < 18.5 (underweight) | 15,285 (4.7) | 6593 (3.9) |
| 18.5 – 24.99 (normal) | 143,104 (44.4) | 69,135 (40.7) |
| 25.0 – 29.99 (overweight) | 45,309 (14.0) | 24,273 (14.3) |
| ≥ 30 (obese) | 25,749 (8.0) | 14,818 (8.7) |
| Missing | 93,163 (28.9) | 54,980 (32.4) |
| **Previous preterm deliveries, n (%)** | 12,473 (3.9) | 6816 (4.0) |
| **Parity, n (%)** |  |  |
| Multiparous | 172,592 (53.5) | 92,007 (54.2) |
| Nulliparous | 149,997 (46.5) | 77,785 (45.8) |
| Unknown | 21 (0.0) | 7 (0.0) |
| **Diabetes, n (%)** |  |  |
| Any type of diabetes prior to delivery | 26,872 (8.3) | 12,451 (7.3) |
| Gestational diabetes | 25,452 (7.9) | 11,677 (6.9) |
| Diabetes pre-pregnancy | 1420 (0.4) | 774 (0.5) |
| **Hypertension, n (%)** |  |  |
| Pregnancy induced hypertension | 15,786 (4.9) | 9278 (5.5) |
| Hypertension due to other causes | 9889 (3.1) | 5256 (3.1) |
| **Antenatal care, mean (SD)** |  |  |
| Number of antenatal visits | 9.29 (3.18) | 9.35 (3.06) |
| Prior hospital admission | 0.13 (0.51) | 0.14 (0.53) |
| **Mode of delivery, n (%)** |  |  |
| Emergency Caesarean section | 61,127 (18.9) | 32,344 (19.0) |
| Elective Caesarean section | 36,009 (11.1) | 19,617 (11.5) |
| Spontaneous vaginal birth | 190,036 (58.9) | 100,176 (59.0) |
| Instrumental vaginal birth | 35,438 (11.0) | 17,662 (10.4) |
| **Induced labour, n (%)** | 65,995 (20.5) | 36,389 (21.4) |
| **Antibiotics used during labour and delivery, n (%)** | 134,020 (41.5) | 67,814 (39.9) |
| **Biological sex, n (%)** |  |  |
| Male | 165,702 (51.4) | 87,099 (51.3) |
| Female | 156,899 (48.6) | 82,698 (48.7) |
| Other | <5 (0.0) | <5 (0.0) |
| Unknown | <5 (0.0) | <5 (0.0) |
| **Year of birth** |  |  |
| 2001-2003 | 78007 (24.2) | 38758 (22.8) |
| 2004-2006 | 70742 (21.9) | 47972 (28.2) |
| 2007-2009 | 84690 (26.2) | 43887 (25.8) |
| 2010-2012 | 89171 (27.6) | 39182 (23.1) |
| **Gestational age at birth, weeks, mean (SD)** | 38.66 (1.99) | 38.68 (1.90) |
| **Admission to NICU, n (%)** | 18,975 (5.9) | 8418 (5.0) |
| **Baby congenital malformation, n (%)** | 16,054 (5.0) | 7917 (4.7) |
| **Small for gestational age, n (%) ^b^** | 22,282 (6.9) | 11,129 (6.6) |
| **Large for gestational age, n (%) ^c^** | 40,736 (12.6) | 22,434 (13.2) |
| **Baby birth weight, mean (SD)** | 3398.17 (581.66) | 3415.70 (566.77) |
| **Apgar 5 scores less than 7, n (%)** | 5105 (1.6) | 2441 (1.4) |

* Abbreviations: *BMI* body mass index, *NICU* neonatal intensive care unit

^a^ Equal to weight (kilograms) divided by height (meters) squared

^b^ Below the 10^th^ percentile of weight for final GA and biological sex

^c^Above the 90^th^ percentile of weight for final GA and biological sex

**Supplementary Table 4.** Multivariable logistic regression models: Prenatal and preconception mental health and subdomain vulnerability

|  | No prenatal depression- episodic preconception history | | No prenatal depression- persistent preconception history | | Prenatal depression- no preconception history | | Prenatal depression- episodic preconception history | | Prenatal depression- persistent preconception history | |
| --- | --- | --- | --- | --- | --- | --- | --- | --- | --- | --- |
| **Developmental domain** | Crude OR (95% CI)^a^ | Adjusted OR (95% CI) ^a,b^ | Crude OR (95% CI)^a^ | Adjusted OR (95% CI) ^a,b^ | Crude OR (95% CI)^a^ | Adjusted OR (95% CI) ^a,b^ | Crude OR (95% CI)^a^ | Adjusted OR (95% CI) ^a,b^ | Crude OR (95% CI)^a^ | Adjusted OR (95% CI) ^a,b^ |
| Physical health and well-being | 1.25  (1.20-1.30) | **1.19**  **(1.14-1.23)** | **1.55**  **(1.48 -1.63)** | **1.40**  **(1.33-1.48)** | 1.14  (0.94-1.37) | 1.04  (0.86 - 1.26) | **1.67**  **(1.49 - 1.88)** | **1.41**  **(1.24 - 1.59)** | **2.16**  **(1.98 - 2.37)** | **1.74**  **(1.57 – 1.93)** |
| Social competence | **1.20**  **(1.16-1.25)** | **1.18**  **(1.13-1.22)** | **1.44**  **(1.37 -1.51)** | **1.38**  **(1.31 -1.46)** | **1.35**  **(1.14 -1.61)** | **1.27**  **(1.07 - 1.52)** | **1.53**  **(1.36 - 1.72)** | **1.36**  **(1.20 - 1.55)** | **1.95**  **(1.78 - 2.14)** | **1.71**  **(1.53 - 1.90)** |
| Emotional maturity | **1.21**  **(1.16-1.25)** | **1.18**  **(1.13-1.22)** | **1.41**  **(1.35 -1.48)** | **1.33**  **(1.27-1.46)** | **1.29**  **(1.08-1.53)** | **1.22**  **(1.02 - 1.46)** | **1.35**  **(1.20 - 1.52)** | **1.22**  **(1.07 - 1.38)** | **1.95**  **(1.78 - 2.14)** | **1.67**  **(1.51 - 1.86)** |
| Language and cognitive  development | **1.23**  **(1.18-1.29)** | **1.18**  **(1.12-1.23)** | **1.42**  **(1.34-1.50)** | **1.32**  **(1.24-1.40)** | **1.29**  **(1.04-1.58)** | 1.19  (0.96 - 1.47) | **1.47**  **(1.28 - 1.69)** | **1.23**  **(1.06 - 1.43)** | **1.72**  **(1.54 - 1.93)** | **1.46**  **(1.28 - 1.66)** |
| Communication skills and  general knowledge | 0.98  (0.94-1.01) | **0.94**  **(0.90-0.98)** | 1.02  (0.97 -1.08) | 0.95  (0.90-1.00) | 1.15  (0.96-1.38) | 1.08  (0.89 - 1.30) | 1.09  (0.95 - 1.24) | 0.96  (0.84 - 1.10) | **1.19**  **(1.07 - 1.33)** | 1.04  (0.92 - 1.18) |
| ≥ 1 domain | **1.14**  **(1.11- 1.18)** | **1.11**  **(1.08-1.14)** | **1.33**  **(1.28 -1.38)** | **1.25**  **(1.20-1.30)** | **1.20**  **(1.05-1.38)** | 1.13  (0.98 - 1.30) | **1.34**  **(1.22 - 1.47)** | **1.18**  **(1.06 - 1.30)** | **1.77**  **(1.64 - 1.91)** | **1.51**  **(1.39- 1.65)** |
| ≥ 2 domains | **1.20**  **(1.16-1.24)** | **1.16**  **(1.12-1.20)** | **1.44**  **(1.37 - 1.50)** | **1.35**  **(1.28 - 1.41)** | **1.29**  **(1.09 - 1.52)** | **1.20**  **(1.01 - 1.42)** | **1.49**  **(1.33 - 1.66)** | **1.29**  **(1.15 - 1.46)** | **1.87**  **(1.71 - 2.05)** | **1.59**  **(1.44 – 1.77)** |

^a^ Values in bold indicate significance at p < 0.05; ^b^Adjusted for **the pregnant person’s characteristics at conception** (the pregnant person’s age at the time of conception and income quintile), child biological sex, year of birth, nulliparity, pre-existing diabetes, pre-conception BMI and **medication use** (1-year preconception use of antidepressants, antipsychotics, and anxiolytics)

Note: A child is deemed to be vulnerable on a particular domain if their scores are in the bottom 10% using BC-Provincial cut-offs

Note: Reference group refers to those without prenatal depression and without diagnosed depression and/or anxiety 0-3 years preconception (n=66652)

Note: This table is based on the analysis conducted after performing listwise deletion of missing data.
